# Decitabine shows anti-acute myeloid leukemia potential via regulating the miR-212-5p/CCNT2 axis

**DOI:** 10.1515/biol-2020-0097

**Published:** 2020-12-31

**Authors:** Lina Xing, Jinhai Ren, Xiaonan Guo, Shukai Qiao, Tian Tian

**Affiliations:** Department of Hematology, The Second Hospital of Hebei Medical University, No. 215 Hepingxi Road, Xinhua District, Shijiazhuang City, 050000, Hebei Province, China

**Keywords:** decitabine, miR-212-5p, AML, CCNT2

## Abstract

Previous research has revealed the involvement of microRNA-212-5p (miR-212-5p) and cyclin T2 (CCNT2) in acute myeloid leukemia (AML). However, whether the miR-212-5p/CCNT2 axis is required for the function of decitabine in AML has not been well elucidated. Quantitative reverse transcription-polymerase chain reaction was used to examine enrichment of miR-212-5p. The relationship between CCNT2 and miR-212-5p was verified by the luciferase reporter assay. Cell apoptosis was evaluated by flow cytometry and western blot. CCK-8 assay was performed to determine cell viability. Decitabine significantly repressed cell viability, while promoted cell apoptosis. Meanwhile, the expression levels of cyclinD1, CDK4, and Bcl-2 were suppressed in cells with decitabine exposure, but Bax and caspase-3 expression levels were upregulated. Besides, miR-212-5p upregulation had the similar function with decitabine in AML cell proliferation and apoptosis. Subsequently, restoration of CCNT2 attenuated miR-212-5p overexpression-induced effects in Kasumi-1 and SKNO-1 cells. In addition, miR-212-5p depletion reversed decitabine-induced CCNT2 downregulation. The miR-212-5p/CCNT2 axis had an implication in the anti-leukemic effect of decitabine in AML.

## 1 Introduction

Acute myeloid leukemia (AML) is a malignant leukemia affecting adults [1,2]. Although great progression in AML treatment has been made, the prognosis and outcome of AML remain dismal.

Many studies have shown that hypomethylating agents contribute to the treatment of hematologic malignancies, including AML [3,4,5]. Decitabine, a DNA hypomethylating agent, is one of the most commonly utilized therapeutics for AML [6]. In AML cell lines, it has been observed that decitabine inhibits cell viability and promotes apoptosis [7]. However, the effect of decitabine is still limited. MicroRNAs (miRNAs) are widely reported to modulate cellular viability, survival, as well as differentiation, and act as the potential therapeutic targets for AML [8]. For instance, Yan et al. demonstrated low expression of miR-217 in AML *in vivo*, which may be a prognostic indicator for AML [9]. In another report, loss of miR-345-5p promoted proliferation and blocked apoptosis by targeting AKT2 in AML [10]. Previous studies pointed out that miR-29b overexpression could increase the antileukemic activity of decitabine [11,12]. Low expression of miR-29a was linked to the malignant progression in pediatric AML patients [13]. These findings supported the important roles of miRNAs in predicting prognosis of AML. More importantly, miRNAs may have the potential to synergize with decitabine to treat AML. Previous studies have revealed the protective role of miR-212-5p in various diseases, including AML [14,15]. These results further showed that miR-212-5p could regulate the proliferation and apoptosis of AML *in vitro* [15]. Cyclin T2 (CCNT2) is required for the RNA polymerase II-mediated transcription process [16,17]. More importantly, a previous investigation indicated that miR-192 suppressed cell viability and cell cycle progression as well as enhanced cell apoptosis in AML by targeting CCNT2 [18]. Although several findings have indicated the roles of miRNAs (including miR-212-5p) and CCNT2 in AML, the involvement of the miR-212-5p/CCNT2 pathway in the antileukemic activity of decitabine has not been fully understood.

In this study, we sought to address whether the miR-212-5p/CCNT2 pathway is required for the antiproliferative function of decitabine in AML.

## 2 Materials and methods

### 2.1 Cell culture and transfection

Cells (Kasumi-1 and SKNO-1 cell lines) were purchased from American Type Culture Collection (Manassas, VA, USA) and maintained in RPMI 1640 (Thermo Fisher Scientific, Waltham, MA, USA) containing 10% fetal bovine serum. CCNT2 overexpression plasmid (pc-CCNT2), pcDNA 3.0 vector (pc-NC), miR-212-5p mimic (agomiR-212-5p), negative control mimic (Scramble), miR-212-5p inhibitor (antagomiR-212-5p), and negative control inhibitor (antagomiR-NC) were constructed. In brief, 1 × 10^6^ cells were plated into a 6-well plate, then the constructs were transfected into the cells using Lipofectamine 3000 (Thermo Fisher Scientific) following the manufacturer’s instruction. 24-72 hours upon transfection, the transfected cells were harvested for subsequent research.

### 2.2 Luciferase reporter assay

CCNT2 fragments including the wild-type (WT-CCNT2) or mutated (MUT-CCNT2) were cloned into the psiCHECK-2 vector. Cells were co-transfected with agomiR-212-5p or Scramble and WT-CCNT2 or MUT-CCNT2 using Lipofectamine 3000. The relative luciferase activity was quantified with the dual-luciferase reporter assay system (Promega, Madison, WI, USA).

### 2.3 Quantitative reverse transcription-polymerase chain reaction (qRT-PCR) assay

Total RNA was extracted using Trizol reagent (Invitrogen, Carlsbad, CA, USA) and DNase I and quantified with Nanodrop 2000 (Thermo Fisher Scientific). Then, reverse transcription was performed using PrimeScript RT Reagent Kit (Takara, Dalian, China). qPCR was conducted using SYBR Green PCR Master Mix (BioSystems, Foster City, CA, USA). Beta-actin (β-actin) was employed as the internal control, which was the most stable reference gene in AML analyzed by GeNorm and NormFinder [19,20]. Relative expression levels of CCNT2 and miR-212-5p were calculated using the 2^−ΔΔCt^ method. The primer sequences are presented in Table 1.

**Table 1:** The primers used in qRT-PCR

Gene	Primer sequence	*T*_m_ (°C)	Amplification efficiency (%)	Product size (bp)
miR-212-5p	F: ACCTTGGCTCTAGACTGCT	57.6	92.30	74
	R: GCAGGGTCCGAGGTATTC	56.8		
CCNT2	F: GGAGTGGAGGCGGATAAAGAG	59.9	94.70	84
	R: AGAGACATTGAGACGCTGTCC	59.8		
U6	F: TAGGATTATACATTGTAAGAGGT	51.8	93.50	119
	R: GTGTGCTACAGAATTTAAAGGTT	55.4		
β-Actin	F: CTTCGCGGGCGACGAT	59.9	96.40	104
	R: CCACATAGGAATCCTTCTGAC	58.7		

### 2.4 Western blot

Total protein was extracted from cells using RIPA lysis buffer (Beyotime, Shanghai, China) containing phenylmethylsulfonyl fluoride (PMSF; Beyotime) and separated by sodium dodecyl sulfate-polyacrylamide gel electrophoresis. Samples were then transferred to polyvinylidene difluoride membranes (Millipore, Bedford, MA, USA). Afterward, the membranes were blocked with 5% skim milk for 1 h at room temperature. The membranes were incubated with the following primary antibodies at 4°C overnight: anti-CCNT2 (1:1,000; ab96133; Abcam, Cambridge, UK), anti-β-actin (1:2,500; ab8227; Abcam), anti-cyclinD1 (1:5,000; ab134175; Abcam), anti-CDK4 (1:1,000; ab108357; Abcam), anti-Bcl-2 (1:1,000; ab32124; Abcam), anti-Bax (1:1,000; ab32503; Abcam), and anti-caspase-3 (1:500; ab13847; Abcam); and then incubated with secondary antibody (1:5,000; ab205718; Abcam) for 1 h at room temperature. Protein bands were visualized using ECL kits (Thermo Fisher Scientific).

### 2.5 Cell Counting Kit-8 (CCK-8) assay

After decitabine (Sigma-Aldrich, Germany) treatment and/or miRNA and plasmid transfection, cells at a density of 5–6 × 10^5^ per well (96-well plate) in 100 µL of culture medium were tested. Ten microliters of the CCK-8 solution (Beyotime) were added into each well and incubated for 4 h. Absorbance was measured at 450 nm using a microplate reader (Bio-Rad, Hercules, CA, USA).

### 2.6 Cell apoptosis assay

The number of cells per sample was 1–5 × 10^6^/mL. The cells were washed once with incubation buffer, resuspended with 100 µL of labeling solution (labeling solution: Annexin V-FITC and PI in the incubation buffer at a final concentration of 1 µg/mL), incubated for 15 min at room temperature, and collected by centrifugation at 1,000 rpm for 5 min. Apoptotic cells were detected using a flow cytometer (BD Biosciences, San Jose, CA, USA).

### 2.7 Statistical analysis

Statistical analysis in this study was performed using Graphpad 7.0 statistical software (Graphpad, San Diego, CA, USA). The differences were determined using Student’s *t*-test or one-way ANOVA. *P* values less than 0.05 were considered statistically significant.

## 3 Results

### 3.1 Decitabine decreases proliferation while promotes apoptosis of AML cells

**Figure 1:**
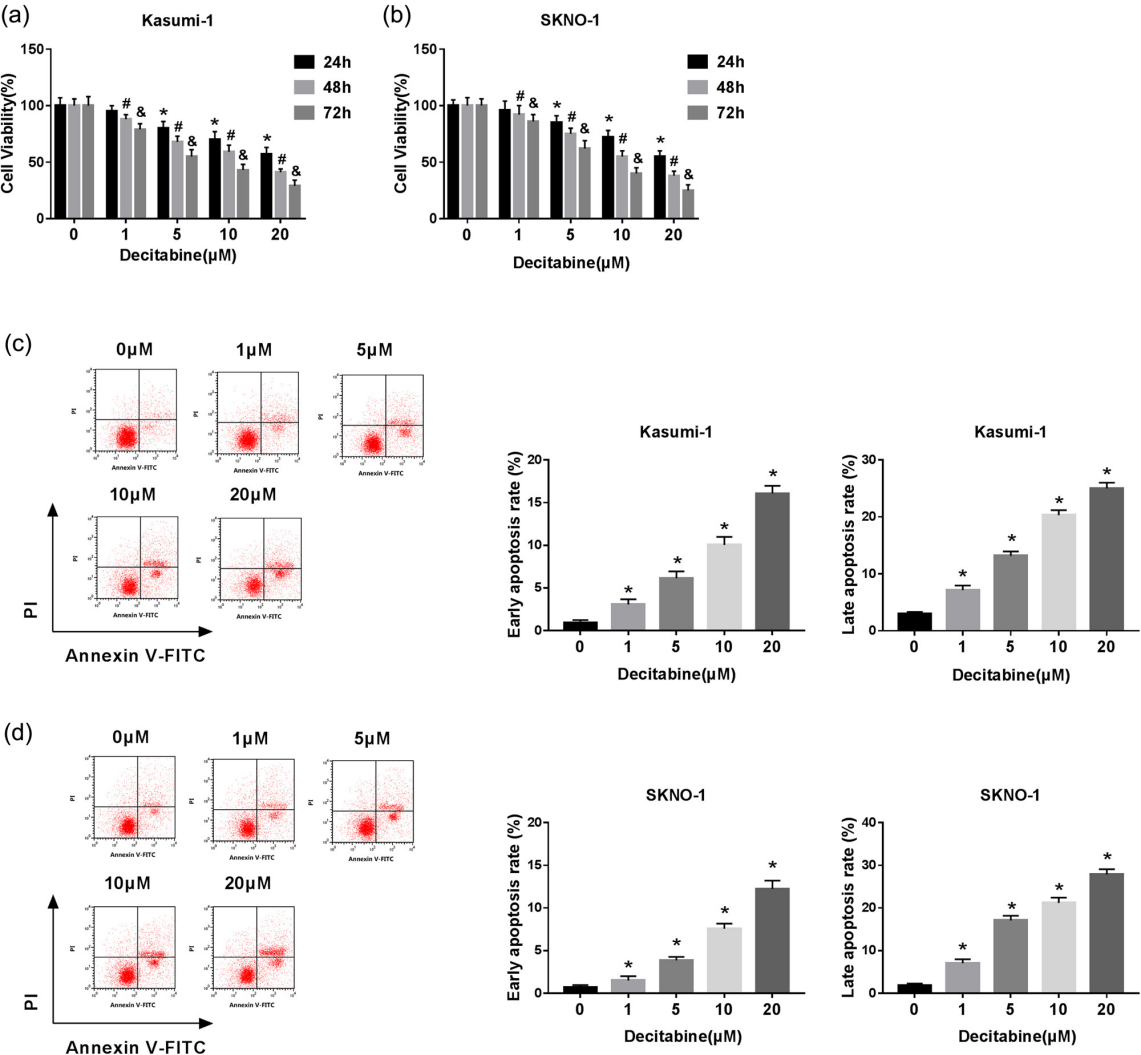
Effect of decitabine on the viability and apoptosis of AML cells. (a and b) CCK-8 assay was used to determine the viability of cells treated with various concentrations of decitabine. (c and d) Cell apoptosis was evaluated by flow cytometry. *P < 0.05 compared with 24 h treatment; #*P* < 0.05 compared with 48 h treatment; &*P* < 0.05 compared with 72 h treatment.

**Figure 2:**
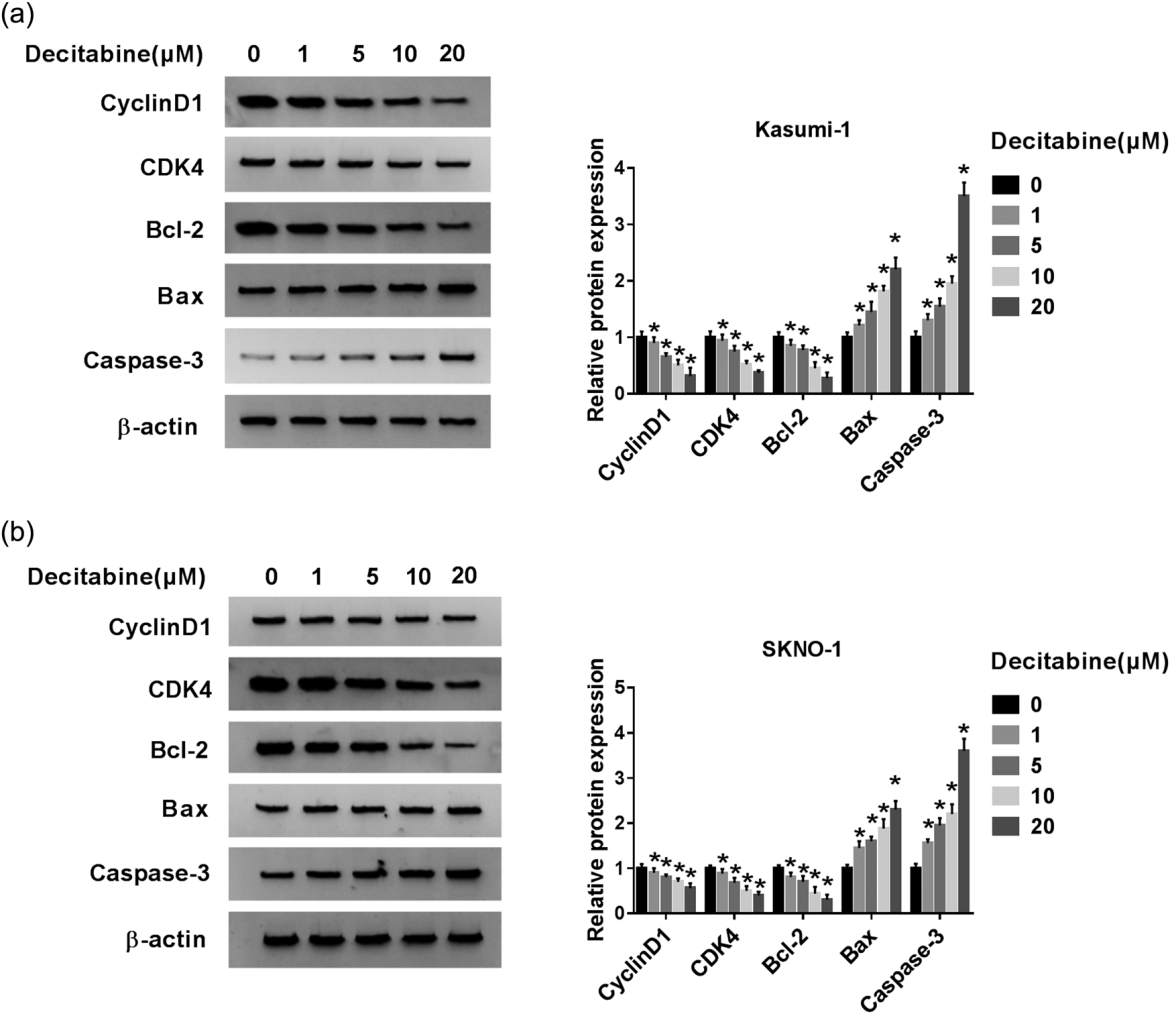
Effect of decitabine on the expression levels of proliferation- and apoptosis-related proteins. (a and b) Western blot was performed to examine the protein levels of cyclinD1, CDK4, Bcl-2, Bax, and caspase-3 in Kasumi-1 and SKNO-1 cells after treatment with different concentrations of decitabine. *P < 0.05.

**Figure 3:**
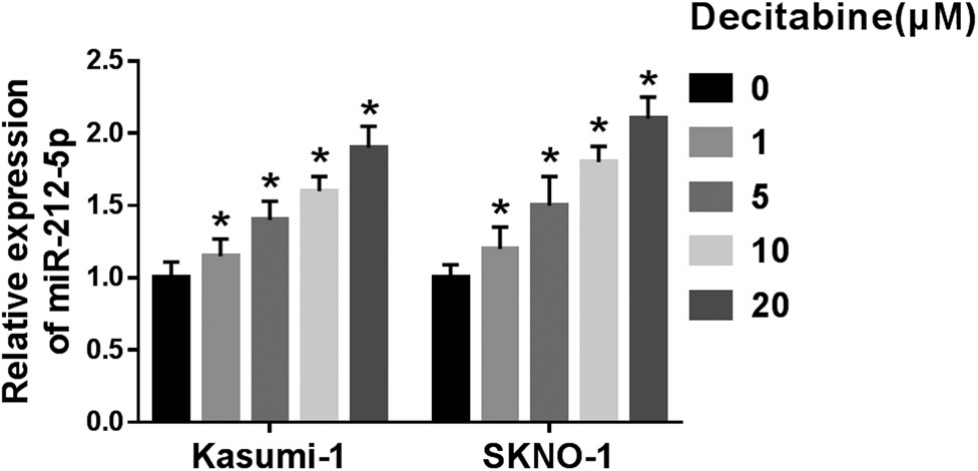
Effect of decitabine on miR-212-5p expression level in Kasumi-1 and SKNO-1 cells. Cells were exposed to decitabine, and the level of miR-212-5p was detected using qRT-PCR assay. *P < 0.05.

In this study, decitabine was used to treat AML cells. As shown in Figure 1a and b, various concentrations of decitabine were used to stimulate Kasumi-1 and SKNO-1 cells. Our data indicated that cell viability was significantly inhibited after treatment with different concentrations of decitabine. The results of flow cytometry further outlined that decitabine notably increased the cell apoptotic rate (Figure 1c and d). To expound the molecular basis of these processes, the expression levels of proliferation/apoptosis-related proteins were detected (Figure 2a and b). We found that cyclinD1 and CDK4, proliferation-related proteins, were decreased in decitabine-treated cells. Meanwhile, Bcl-2 expression was also downregulated. In contrast, the levels of Bax and caspase-3 were distinctly upregulated in cells exposed to decitabine. Decitabine at a concentration of 5 µM was selected for the following experiments. MiR-212-5p was significantly upregulated in decitabine-treated cells (Figure 3). These data implied that miR-212-5p may be related to the function of decitabine in AML cells.

### 3.2 MiR-212-5p upregulation decreases cell viability, while increases cell apoptosis

**Figure 4:**
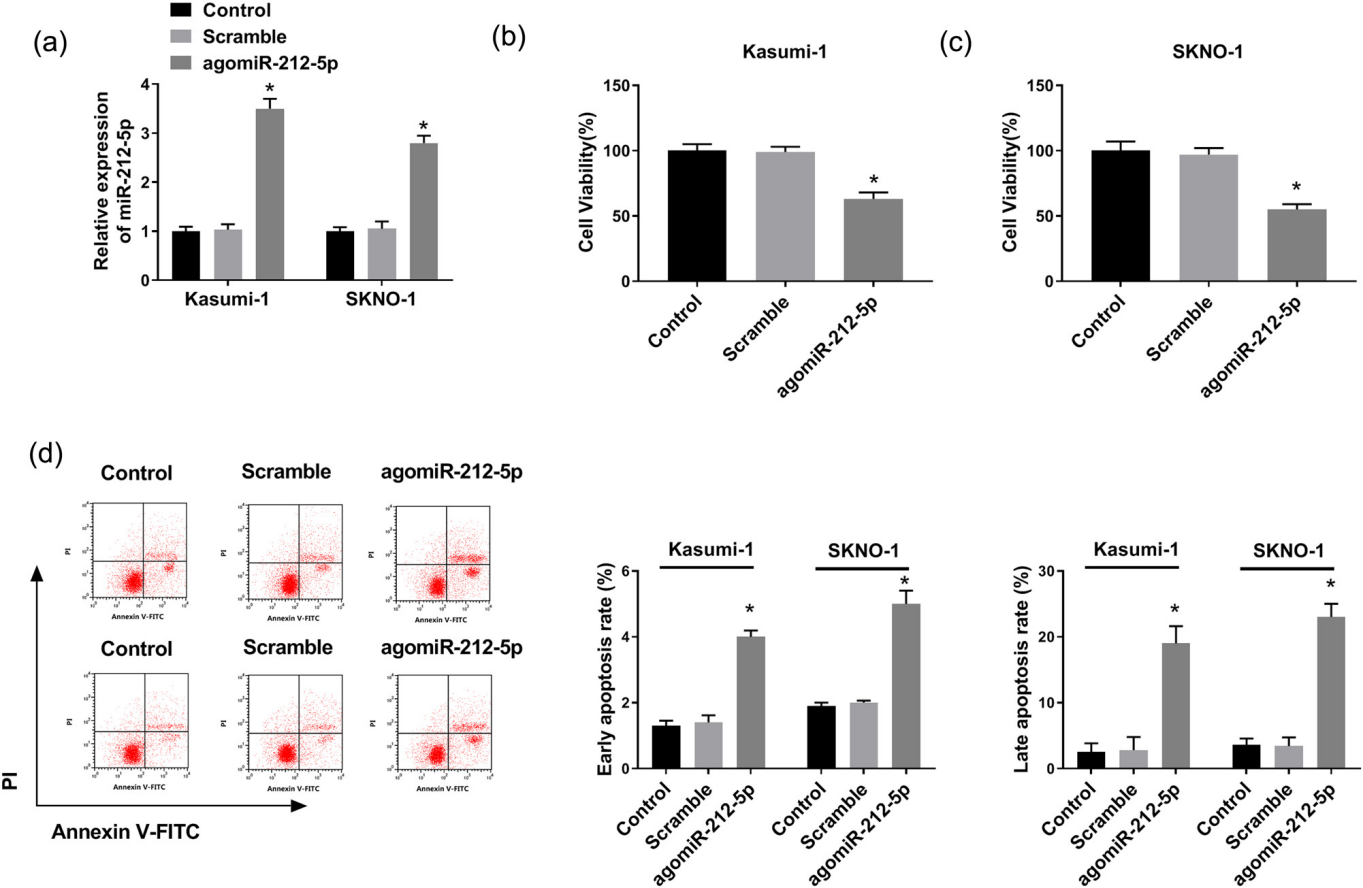
Effect of miR-212-5p overexpression on cell viability and apoptosis. (a) qRT-PCR was carried out to evaluate miR-212-5p expression in cells transfected with Scramble or agomiR-212-5p. (b and c) CCK-8 assay was employed to determine cell viability. (d) Cell apoptotic rate was examined in Kasumi-1 and SKNO-1 cells of each group. *P < 0.05.

**Figure 5:**
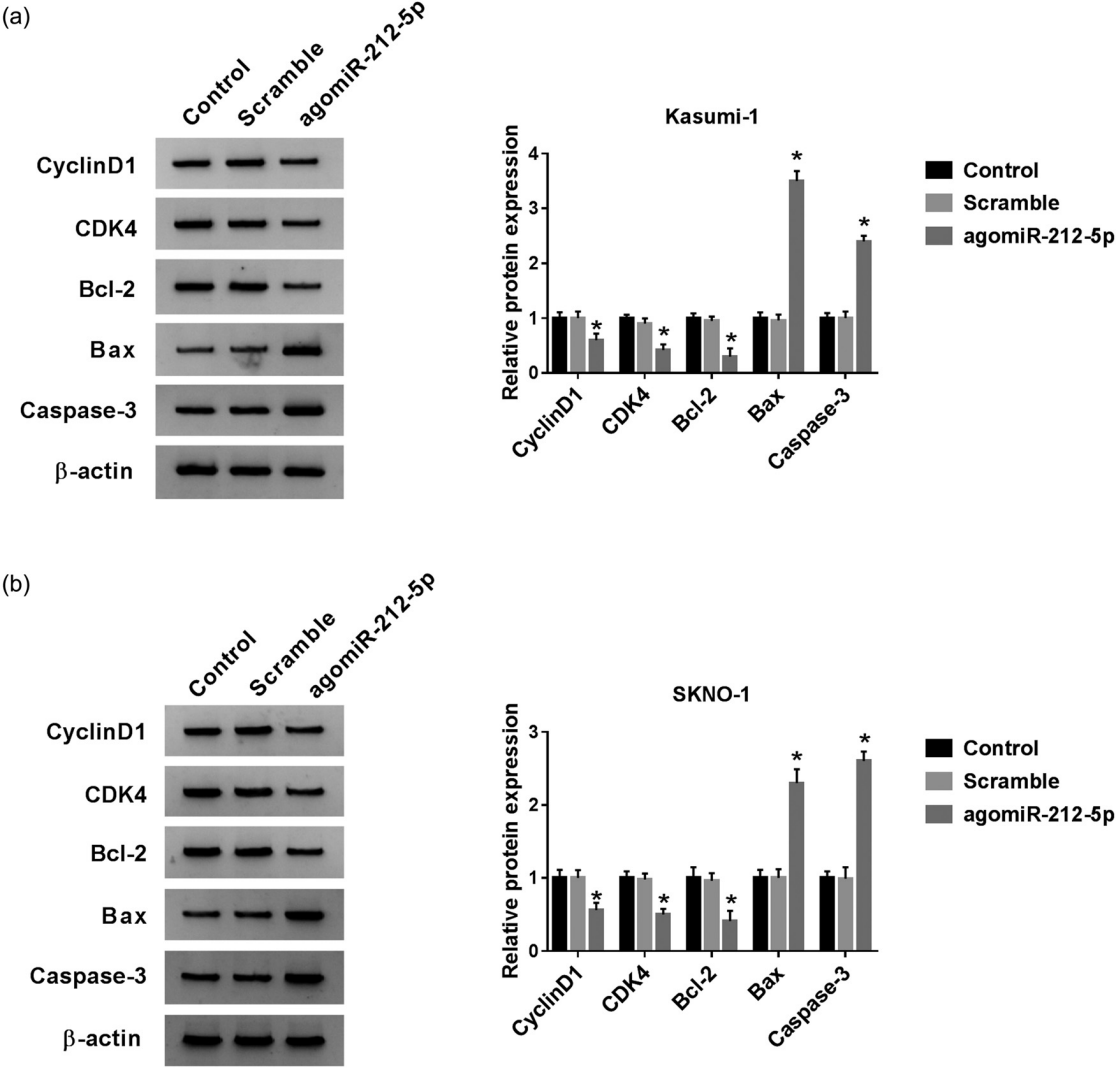
Effect of miR-212-5p upregulation on the expression levels of proliferation- and apoptosis-related proteins in AML cells. (a and b) The protein levels of cyclinD1, CDK4, Bcl-2, Bax, and caspase-3 in cells transfected with agomiR-212-5p. *P < 0.05.

Then, we upregulated miR-212-5p expression in AML cells using agomiR-212-5p. The transfection efficiency demonstrated that the introduction of agomiR-212-5p distinctly upregulated the miR-212-5p level (Figure 4a). The agomiRNA-mediated overexpression of miR-212-5p inhibited cell viability (Figure 4b and c). In addition, the cell apoptotic rate was remarkably increased by miR-212-5p overexpression (Figure 4d). Simultaneously, the result of the western blot analysis of proliferation/apoptosis-related proteins was also consistent with that of CCK-8 and flow cytometry assays (Figure 5a and b), further confirming that the gain of miR-212-5p may participate in decitabine-induced proliferation suppression and apoptosis promotion in AML cells.

### 3.3 MiR-212-5p contains the binding sites with CCNT2

**Figure 6:**
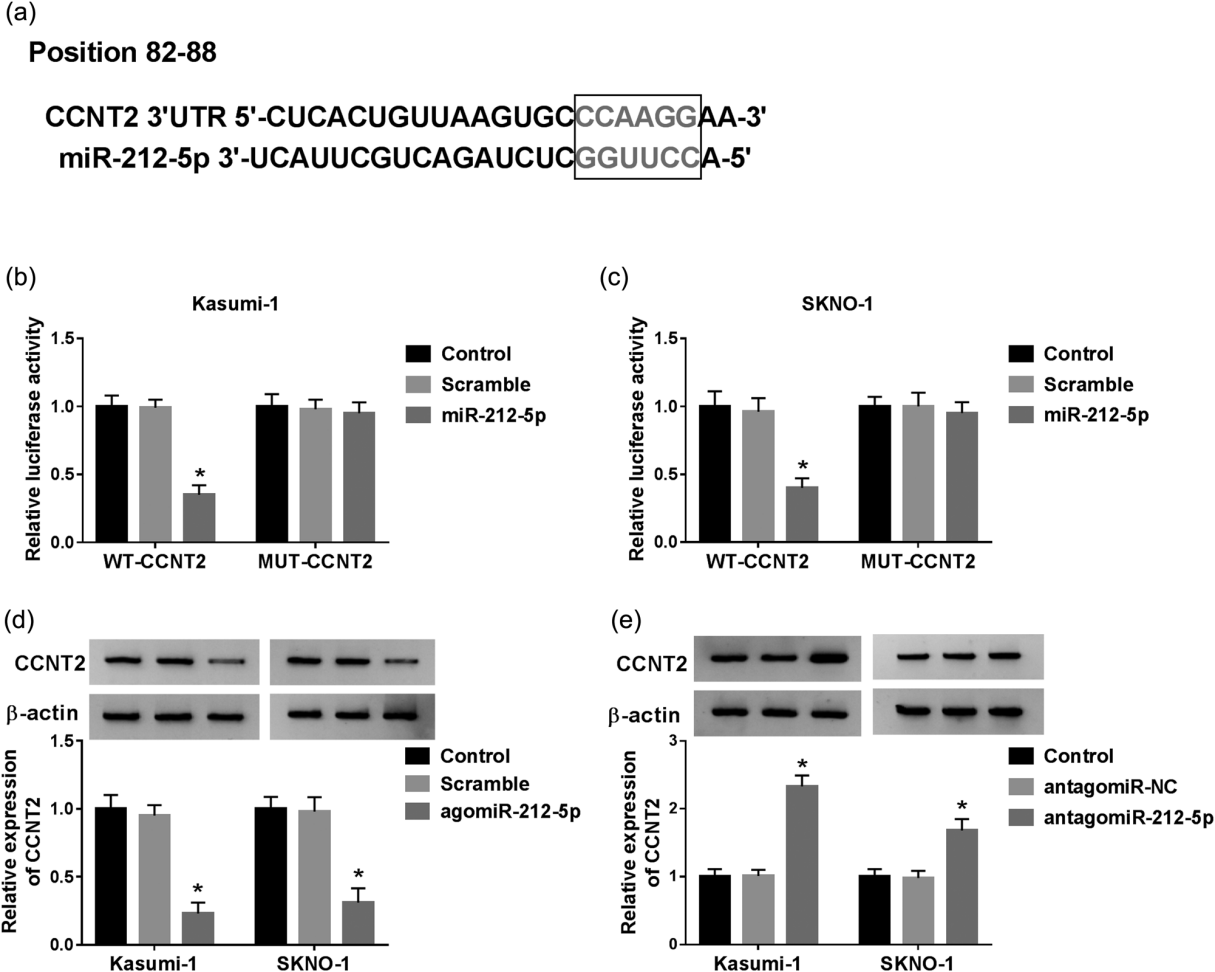
MiR-212-5p interacts with CCNT2. (a) The complementary sequences of miR-212-5p and CCNT2 were predicted by TargetScan online database. (b and c) AgomiR-212-5p or Scramble and MUT-CCNT2 or WT-CCNT2 were transfected into Kasumi-1 and SKNO-1 cells, and then, the luciferase activity was examined. (d) Western blot was performed to detect the protein level of CCNT2 in cells introduced with agomiR-212-5p or Scramble. (e) The relative expression of CCNT2 in antagomiR-212-5p- or antagomiR-NC-transfected cells. *P < 0.05.

It is well known that miRNAs regulate cellular processes by repressing target genes. We searched the potential target gene of miR-212-5p using an online tool TargetScan. The data uncovered that miR-212-5p harbored binding sequences complementary to CCNT2 seed regions (Figure 6a). We further addressed whether miR-212-5p targets CCNT2. As a result, the transfection of agomiR-212-5p greatly decreased the luciferase activity of the WT-CCNT2 group, while no obvious change was observed in the MUT-CCNT2 group (Figure 6b and c). As demonstrated in Figure 6d, the delivery of agomiR-212-5p induced the inhibition of the CCNT2 protein level. However, the interference of miR-212-5p elevated the CCNT2 level (Figure 6e).

### 3.4 Restoration of CCNT2 attenuates the effects of miR-212-5p upregulation in AML

**Figure 7:**
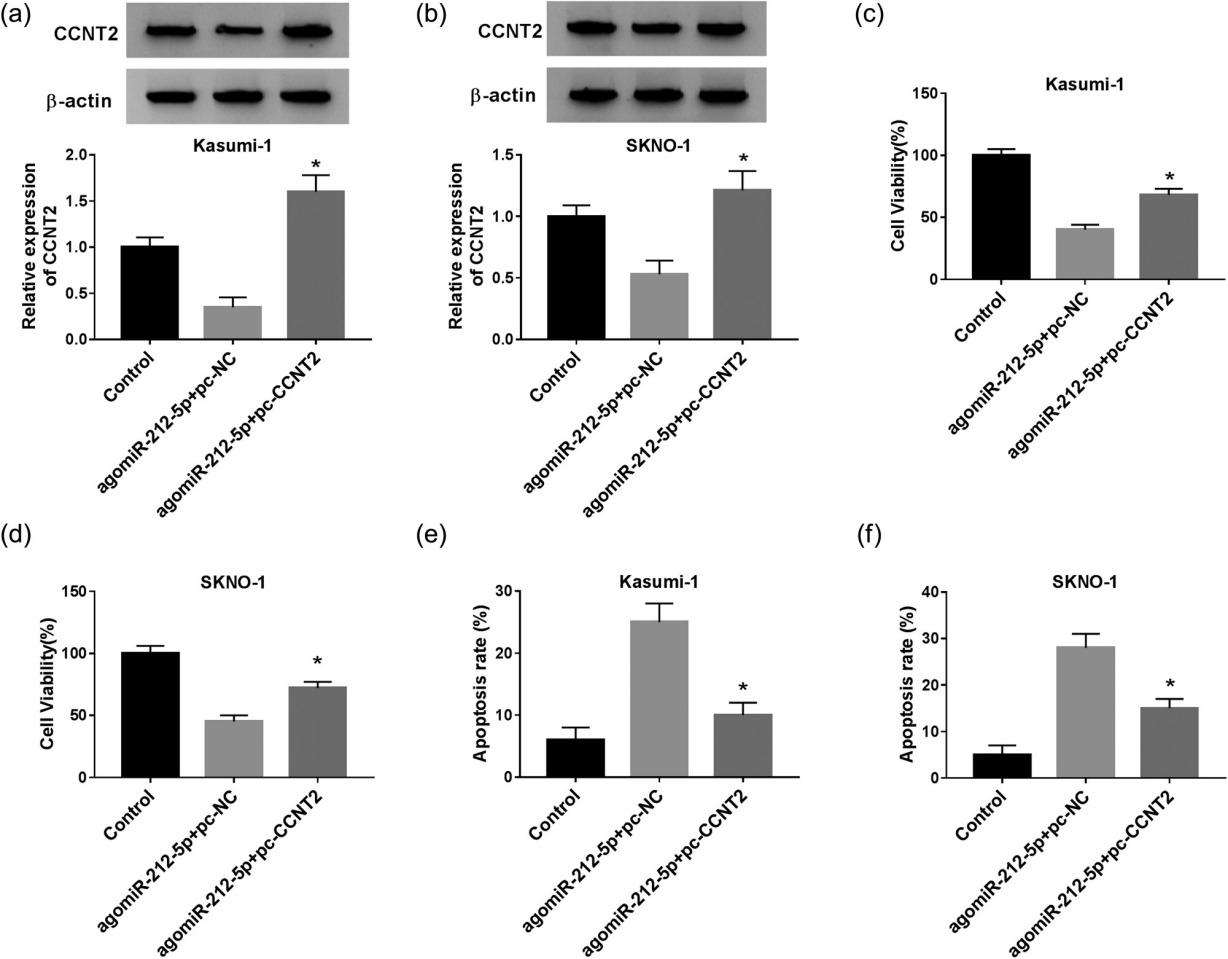
Effect of CCNT2 overexpression on miR-212-5p upregulation-induced effects in AML cells. (a and b) Cells were co-transfected with agomiR-212-5p and CCNT2 overexpression plasmid (pc-CCNT2) and then subjected to western blot analysis of CCNT2. (c–f) Cell viability and apoptosis were determined by CCK-8 assay and flow cytometry, respectively. *P < 0.05.

We performed the rescue-of-function experiment to confirm the involvement of miR-212-5p/CCNT2 axis in the regulation of proliferation and apoptosis of AML. As displayed in Figure 7a and b, the protein level of CCNT2 was lower in miR-212-5p overexpressing cells than that of the control group, while it was rescued by forced expression of CCNT2. CCK-8 assay further indicated that elevated CCNT2 expression regained miR-212-5p upregulation-induced inhibition of cell viability (Figure 7c and d). When compared with the agomiR-212-5p + pc-NC group, cell apoptosis was blocked in the agomiR-212-5p + pc-CCNT2 group (Figure 7e and f). These results revealed that pc-CCNT2 transfection distinctly overturned the impact of miR-212-5p upregulation on cell viability and apoptosis.

### 3.5 MiR-212-5p ablation overturns decitabine-induced CCNT2 downregulation

**Figure 8:**
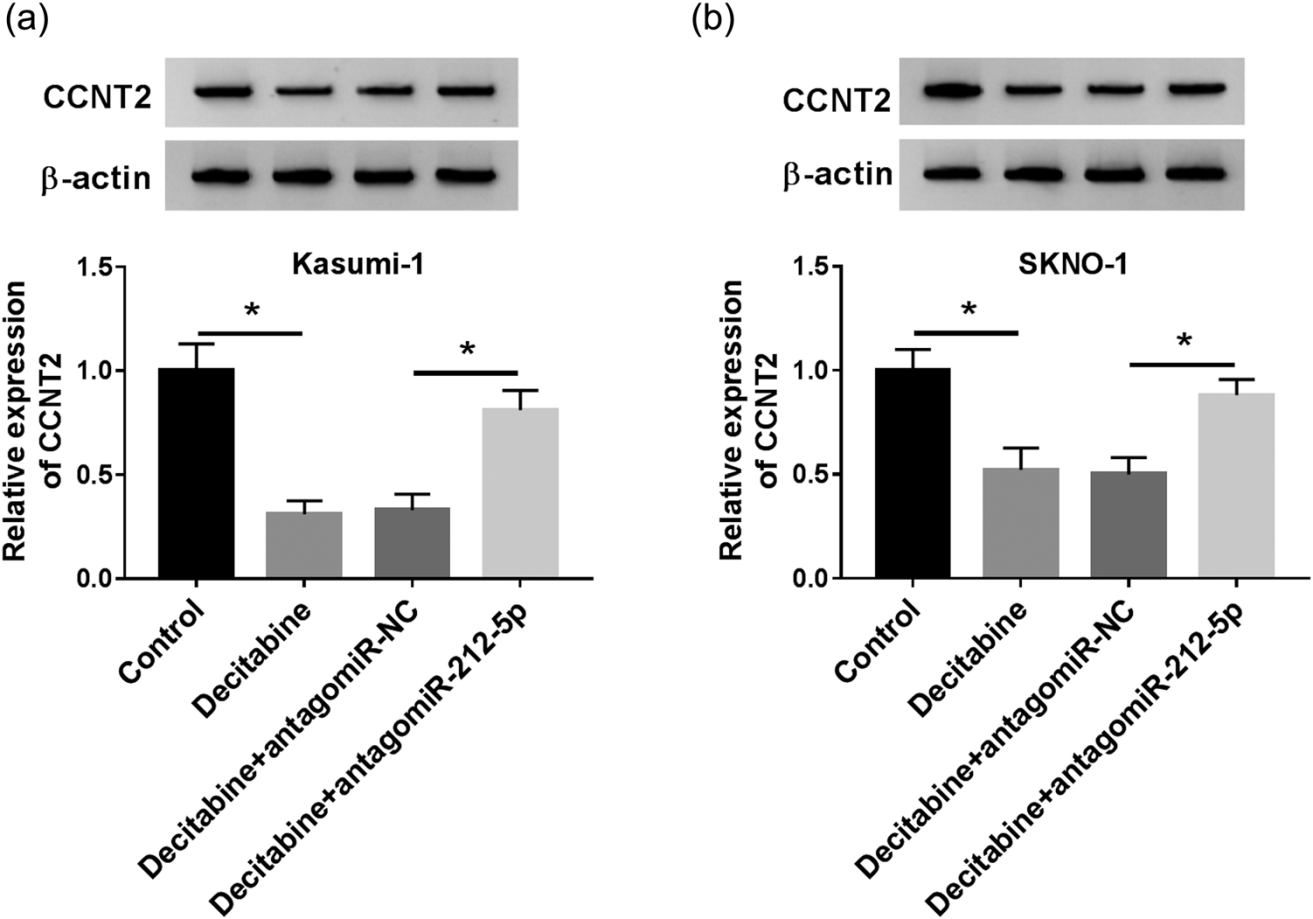
Decitabine regulates the expression of CCNT2 via miR-212-5p. (a and b) Western blot was conducted to evaluate the expression of CCNT2 in cells with decitabine exposure and antagomiR-212-5p transfection. *P < 0.05.

**Figure 9:**
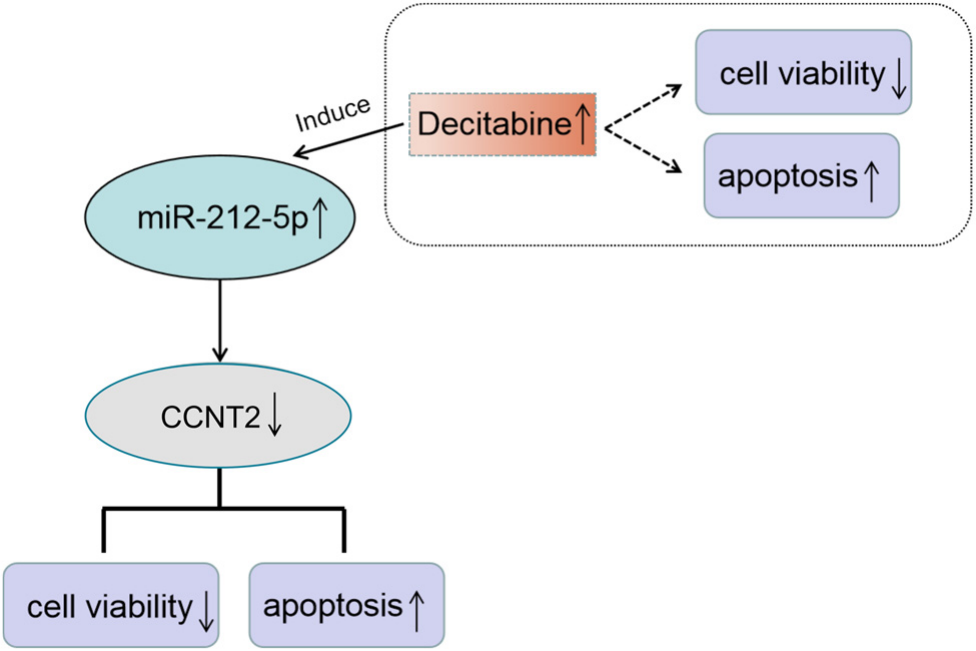
Decitabine inhibits cell proliferation and enhances apoptosis via the miR-212-5p/CCNT2 axis in AML cells.

Finally, the level of CCNT2 in AML cells treated with decitabine and antagomiR-212-5p was also examined using qRT-PCR. In Kasumi-1 cells, we disclosed that the protein level of CCNT2 was inhibited in the decitabine group compared with that in the control group. However, the introduction of antagomiR-212-5p dramatically recuperated the decitabine-induced CCNT2 suppression (Figure 8a). As expected, miR-212-5p ablation overturned decitabine-induced CCNT2 downregulation in SKNO-1 cells (Figure 8b). Collectively, all these data implied that the miR-212-5p/CCNT2 axis may be responsible for the function of decitabine in AML, further demonstrating the potential of miR-212-5p combined with decitabine in AML treatment by inducing cell proliferation inhibition and apoptosis (Figure 9).

## 4 Discussion

AML develops in people of all ages, especially in the elderly, who have poor overall survival rates [2,21]. It has been well documented that decitabine has acceptable efficacy for the treatment of AML [22,23]. However, there are little data about the molecular mechanism of decitabine treatment of AML. Therefore, a deep understanding of the related molecular mechanisms of decitabine in AML may contribute to the treatment of AML. In our study, we also confirmed the anti-AML activity of decitabine via evaluating cell viability and apoptosis. According to the cell viability of Kasumi-1 and SKNO-1 cells treated with different concentrations of decitabine for different times, decitabine at a concentration of 5 µM for 48 h was used to explore the regulatory mechanism of decitabine function in AML.

Accumulating studies supported that many miRNAs participate in the development of various diseases, including AML [8,9]. Emerging evidence has revealed the involvement of miR-212-5p in various diseases [14,24,25]. Researchers have reported that miR-212-5p prevents dopaminergic neuron death in the mouse model of Parkinson’s disease [24]. In another study, high level of miR-212-5p blocks epithelial–mesenchymal transition in breast cancer, thereby inhibiting cancer progression [26]. A study also revealed that downregulation of miR-212-5p is related to MIF-AS1-mediated promotion of proliferation and apoptosis inhibition in gastric cancer [27]. Recently, the previous study has demonstrated that the expression of miR-212-5p is low in AML patients and cells. They further indicated that the upregulation of miR-212-5p blocks cell viability and proliferation, while increases apoptosis in Kasumi-1 cells [15]. Consistently, we disclosed that the miR-212-5p level was elevated in cells exposed to different concentrations of decitabine. Moreover, gain of miR-212-5p induced cell viability promotion, as well as increased the cell apoptosis rate, implying that miR-212-5p may participate in the process of AML response to decitabine. The induced expression of miR-212-5p by decitabine and its role in AML suggested that miR-212-5p might serve as a potential biomarker in AML or in decitabine-treated AML; however, this still needs to be verified by further clinical test.

An increasing number of miRNA–mRNA networks was identified to be associated with AML [28,29]. A previous study stated that miR-142-3p may play a suppressor role in the development of gastric cancer via downregulating CCNT2 [30]. A study confirmed that CCNT2 act as a target of miR-15a to block spermatogenesis [31]. MiR-297c-5p impedes cell proliferation via negatively affecting CCNT2 expression in oligodendrocyte progenitor cells [32]. Recent evidence showed that CCNT2 may act as the target of miR-192 and participate in the progression of AML [18]. But whether CCNT2 is associated with the miR-212-5p-mediated function has not been elaborated. Interestingly, results from this study provided the evidence that CCNT2 contained the binding sites with miR-212-5p and the expression of CCNT2 could be impeded by miR-212-5p, referring that CCNT2 may have an implication in the function of miR-212-5p in decitabine-treated cells. Subsequently, in rescue-of-function experiment, the function of miR-212-5p overexpression to inhibit cell viability and promote cell apoptosis was blocked by CCNT2 upregulation in AML, and these results suggested that miR-212-5p effected the behavior of AML cells via targeting CCNT2. In further experiments, CCNT2 was visibly decreased in AML cells treated with decitabine; nevertheless, the decitabine-induced suppression of CCNT2 was relieved by downregulation of miR-212-5p. In summary, these experiments proved that decitabine repressed cell proliferation and enhanced apoptosis via inducing miR-212-5p expression to downregulate CCNT2 in AML (Figure 9).

There are some limitations in this study. It is vital to explore the detailed molecular mechanisms of the miR-212-5p/CCNT2 axis in the treatment of AML. At the same time, the role of CCNT2 alone should be anatomized in future. In addition, whether the miR-212-5p/CCNT2 pathway axis influences other cell biological processes in decitabine-treated AML cells also requires more intensive research. Their function should be addressed *in vivo* in future.

In conclusion, these findings shed light on that miR-212-5p may serve as a potential target of AML therapy. MiR-212-5p/CCNT2 pathway is a novel mechanism of decitabine-induced response in AML cells, which could bridge an important gap in knowledge of decitabine treatment for AML. Meanwhile, our data also provided the theoretical basis for the pathogenesis of AML.
